# Compatibility between Rice Straw Fibers with Different Pretreatments and Ordinary Portland Cement

**DOI:** 10.3390/ma14216402

**Published:** 2021-10-26

**Authors:** Xiaoli Xie, Hongbo Li

**Affiliations:** 1 School of Materials Science and Engineering, Southwest University of Science and Technology, Mianyang 621010, China; 2 Sichuan Zhentong Instection Co., Ltd., Mianyang 621010, China; hongbo_1840@21cn.com

**Keywords:** rice straw fibers, ordinary Portland cement, hydration heat, inhibitory index, lignin, hemicellulose

## Abstract

The compatibility between crop straw and Portland cement greatly restrict the application of crop straw in cement-based materials. In this study, rice straw fibers with different pretreatments were added to ordinary Portland cement (OPC), and the influence of different rice straw fiber (RF) content on the hydration process of OPC was measured using calorimeter tests. Additionally, compatibility between RF and OPC was evaluated using the inhibitory index. As a result, steam explosion treatment of rice straw removed most hemicellulose and post-treatment bleaching was used for delignification. As compared with the pure OPC, addition of RF inhibited the hydration of OPC, and the inhibition degree reduced with the increase in pretreatment degree of RF. The inhibitory index grade of different RF filled OPC (RF-OPC) samples is directly related to hemicellulose and lignin content. Compared with lignin, hemicellulose has a greater influence on cement hydration. Without considering the influence of other components, the RF-OPC samples with hemicellulose content of 1.54 wt.% reached the inhibitory index extreme grade, and the hemicellulose content of 2.05 wt.% led to the cessation of cement hydration. The inhibitory index of the samples with 2.05 and 0.85 wt.% lignin content is moderate and low grade, respectively. In addition, the results of XRD patterns and SEM images are consistent with those of heat of hydration. In terms of mechanical properties of cement-based composites with 10 wt.% rice straw fibers, pretreatment of fibers is beneficial to improving the fracture toughness of the samples.

## 1. Introduction

The application of plant fibers in cement-based materials has attracted great interest of researchers in recent years because of the low cost, high specific modulus and low density compared with synthetic fiber. Page et al. [1] investigated a method for improving the long-term mechanical performance of flax fiber-reinforced cementitious composites by using alternative binders, including ground granulated blast furnace slag, metakaolin and calcium sulfo-aluminate cement. In order to make efficient use of fibers stemming from the alfa plant, which for the most part is found in North Africa and Spain, Ajouguim et al. [2] investigated the impact of alfa fiber morphology on hydration kinetics and mechanical properties of cement mortars. In addition, many studies on the application of other plant fibers in cement-based materials have published, such as sisal fiber studies described by Frazao et al. [3] and Wei et al. [4], a study of banana fiber [5], a study of pine fiber [6] and a study of hemp fiber [7]. Many studies have been carried out on the mechanical properties of plant reinforced cement-based composites, such as cement-based composites reinforced with diss treated with different methods [8], short discrete jute fiber-reinforced cementitious composites [9], hemp-fiber-reinforced cement-based mortar [10], concrete reinforced with oil palm broom fibers [11] and Curauá-fiber-reinforced cement [12]. The above results show that plant-fiber-reinforced cement-based composites can provide extra energy absorption capacity and convert the brittle material to a ductile one. Moreover, addition of plant fibers can also reduce crack propagation [1,13,14]. Ballesteros et al. pointed out that the research and production of cement-based composites reinforced with plant fibers is a meaningful strategy to overcome environmental problems and social economic issues [15].

According to statistics, agricultural residues around the world reached 100 billion tons, and plants accounted for more than 80 percent of the residues [16]. China is a large agricultural country, and amongst agriculture production, crop straw is the main by-product from cereal harvesting. Despite known benefits, the surplus is burnt so that succeeding crops can be sown on clear fields. Burning of the residue results not only in the waste of biological resources but also in emission of poisonous gases causing severe air pollution of the social environment [17]. In recent years, straw has been useful used for biogas production, but its characteristics limit its performance because straw and stover are substrates characterized by a high content of lignocellulosic compounds that limit their use for anaerobic digestion. Consequently, the production of biogas resulting from the anaerobic digestion of straw and the other substrates is low [18]. Straw pretreatment is an effective method to improve organic matter digestibility, such as the enzymatic pretreatment proposed by Ziemin’ski et al. [19]. In addition, due to farmers’ awareness, technical problems and limitations of other materials, biogas production has not been widely used in China.

In view of the above analysis, cement-based composite reinforced with plant fibers provides an effective approach for the utilization of crop straw and sustainable development strategy of building materials, which is of great significance to the increasingly serious energy shortage and environmental pollution problems. However, the compatibility between the plant fibers and Portland cement greatly restrict the application of plant fibers in cement-based materials. On the one hand, the main components of plant fibers affect the hydration of cement and delay the main hydration reactions of cement [20]. On the other hand, the extractives and impurities of the plant fibers greatly affect the hydration reaction equilibrium of cement. Cabral et al. [21] and Onésippe [22] studied the effect of the sugarcane bagasse fibers on cement-based composites and found that this reaction brings challenges to manufacturing. They deemed that hydration processes of Portland cement become more complicated if plant fibers are used in cement-based materials.

Castro et al. [23] evaluated the compatibility degrees among eight kinds of Amazonian hardwoods and Portland cement. They used the inhibitory index to measure the compatibility degree. The results showed that the cement inhibition directly correlated with arabinose content of the samples because the alkali solution of cement paste dissolve the linkage between lignin and hemicellulose/cellulose and produce large amounts of degraded polysaccharides, which generates an inhibitory effect on cement hydration. Ferraz et al. [24] pretreated the coir fibers with cold water, hot water and NaOH and measured the compatibility of cement and coir fibers via cement hydration temperature. The results showed that addition of CaCl_2_ and the pretreatment with NaOH significantly changed the compatibility between cement and the coir fiber.

In this study, we studied the compatibility between rice straw with different pretreatments and ordinary Portland cement (OPC) to provide a theoretical basis for the engineering application of crop straw in cement-based materials. For the above purpose, untreated and pretreated rice straw fibers with different contents were added to OPC. The heat of hydration and *X*-ray diffraction (XRD) of the composite samples filled with different types and various quantities of rice straw fibers were measured over 7 days. In addition, the inhibitory index was used to express the compatibility degree between rice straw fibers with different pretreatments and OPC. The objectives were to assess compatibility between rice straw fibers and ordinary Portland cement as well as provide an academic basis for the application of crop straw in cement materials in the future.

## 2. Materials and Methods

### 2.1. Raw Materials

In this study, rice straw fibers (RFs) were obtained from farms in the Province of Sichuan, China. To observe the influence of the main components of RF involved in the hydration heat of OPC, pristine rice straw fibers (RF1) were pretreated in the laboratory. Firstly, RF1 treated by steam explosion (RF2) were obtained after the steam explosion pretreatment at 2.5MPa with a solid to liquid ratio of 3:1 for 25 min. Then, to remove the lignin of RF2, RF2 were immersed into hydrogen peroxide aqueous solution together with ozone blowing, and the rice straw fibers treated by bleaching once (RF3) were obtained. The same bleaching process produced the rice straw fibers treated with twice bleaching (RF4). The specific pretreatment process and condition of rice straw was described in Reference [25]. The chemical components of RF1, RF2, RF3 and RF4 are provided in Table 1. The chemical compositions of the rice straw fibers were analyzed according to TAPPI Standard Methods, 2009, as described by Jiang et al. [25]. The average values and standard deviation of length and diameter are shown in Table 2, for which the Layida method was used to determine whether data were accepted or omitted.

A Chinese standard Graded P·O 42.5 type Portland cement (OPC) was used in this study, the chemical components of OPC were determined by *X*-ray fluorescence (XRF) as shown in Table 3.

### 2.2. Characterization Analysis of Rice Straw Fibers

The morphological observations of the different rice straw fiber were carried out on an EVO 18 scanning electron microscope (SEM) made by Zeiss In Jena, Germany. The SEM images were obtained by an electronic microscope model with a 20 kV electron beam. FTIR measurements were conducted using a Spectrum One Autoima spectrometer device made by PerkinElmer OPTICS in America. A total of 24 scans were performed for each sample in the range of 400 to 4000 cm^−1^, with a resolution of 0.5 cm^−1^. Bamboo cellulose fibers were mixed with KBr and ground, subsequently being pressed into a tablet for FTIR measurement.

### 2.3. Experimental Procedure and Method

In this study, the quantities of four rice straw fibers replacing the OPC in the composite pastes were 0–10% by weight (wt.%); the mix proportion of the composite samples and the content of the main components are shown in Table 4. Calorimeter tests with the constant water to solid raw materials (*W/S*) ratio of 1.0 were carried out using an isothermal conduction calorimeter (TAM Air) at the temperature of 20 ± 0.1 °C for a period of 7 days. The hydration heat of the reference OPC sample without RF was measured with the same *W/S* ratio and test time. The hydration heat of each formulation was obtained from three individual tests.

According to the relevant literature [20,21,24], the hydration process of the cement-based composite samples filled with different rice straw fibers was measured using the isothermal conduction calorimeter, and the hydration heat of the different samples was detected. Based on the hydration heat (*Q*) of each formulation, we calculated the inhibitory index (*I*) of the rice straw fibers filled with cement-based composite samples (RF-OPC) as the following equation:

(1) $$I=100*(\frac{Q-Q^{\prime }}{Q})(\frac{H^{\prime }-H}{H})(\frac{S-S^{\prime }}{S})$$

where *I* is the inhibitory index of RF-OPC samples, *Q* is the total hydration heat of 7 days of per unit OPC in the reference samples (J/g), *Q’* is the total hydration heat of 7 days of per unit OPC in RF-OPC samples (J/g), *H* is the time to reach the max rate of hydration heat in the reference samples (h), *H’* is the time to reach the max rate of hydration heat in RF-OPC samples (h), *S* is the max hydration heat increment in the reference samples (J/g/h) and *S’* is the max hydration heat increment in RF-OPC samples (J/g/h).

XRD analysis were performed to observe the influence of the rice straw with different pretreatments on the hydration process of OPC. Each test paste consisting of 20 g of sample with *W*/*S* of 1.0 was placed into a plastic bottle under curing conditions of 20 ± 2 °C and humidity exceeding 90% for 3 and 7 days. Then, the samples were sliced from the hardened composite samples and immersed in alcohol for 7 days to stop the hydration until measurement. The composite samples for XRD analysis were dried at a temperature of 60 °C and then ground into powders (≤45 μm). After that, the samples were measured on a Rigaku D/max-1400 diffractometer (copper target, angle range of 5° to 45°, a voltage of 40 kV and a current of 70 mA) with a step interval and scanning speed of 0.02° and 8°/min, respectively.

Additionally, the composite samples filled with 10 wt.% RF were prepared, and pure cement paste was used as reference sample. The composite samples were prepared using a slurry vacuum de-watering technique. Firstly, RFs were stirred by a Joyoung household soybean milk maker for 2.5 min, then OPC was added and stirred at 1500 rpm in a planetary mixer for an additional 2.5 min. After that, the slurry was transferred to a casting box with size of 160 × 40 mm, superfluous water was absorbed by vacuum and then the samples were pressed at 3.2 MPa for 5 min. The samples were cured at a temperature of 20 ± 2 °C and relative humidity exceeding 90% for 7 and 28 days. For each mixture series, six rectangular samples with the size of 160 × 40 × 8 mm were prepared.

The three-point bending tests were performed using a WSM-10kN universal testing machine of SENS company in Shenzhen, China. In the bending tests, the span and deflection rate were 100 mm and 0.5 mm/min, respectively. The ultimate flexural strength (*MOR*) and fracture toughness (*FT*) were measured using the following equations:

(2) $$MOR=\frac{3Pl}{2bd^{2}}$$

(3) $$FT=\frac{FE}{bd}$$

where *P* refers to the maximum load, *l* represents the support span, *b* is the width of the specimen and *d* stands for the thickness of the specimen. Fracture energy was calculated by integration of the load-deflection curve to the point where the load had dropped to 50% of its maximum value. The results were obtained according to the average value of the six samples.

After the bending test, the fracture surface of the samples cured for 7 days was measured using an EVO 18 scanning electron microscope (SEM) made by Zeiss In Jena, Germany at 20 kV accelerating voltage.

## 3. Results and Discussion

### 3.1. Characterization of Rice Straw Fibers with Different Pretreatments

Hemicellulose, lignin and cellulose are the main components of the plant fibers. Cellulose is the essential component of all the plant fibers, and cellulose type determines the mechanical strength of the plant fibers [15]. As a kind of natural lignocellulosic fiber, each unit of rice straw fiber is composed of crystalline cellulose surrounded and cemented together with hemicellulose and lignin [26]. As shown in Table 1, with the increase in the pretreatment degree, hemicellulose and lignin content gradually decrease, while the content of cellulose gradually increases. The increase in pretreatment degree of RF increases cellulose content, which is 33.6, 50.6, 67.4 and 78.5 wt.% in RF1, RF2, RF3 and RF4, respectively because the pretreatment of straw fiber results in dissolution and reduction in the content of other components, such as hemicellulose, lignin and ash impurities. Steam explosion treatment greatly reduced the content of the hemicellulose component, which reduced to 7.9 wt.% in RF2 from 20.5 wt.% in RF1. Steam explosion pretreatment led to the autohydrolyzation and dissolution of the hemicelluloses and partial depolymerization of lignin by breaking the ether bonding to hemicelluloses [27,28]. Further bleaching treatment seemed to have little effect on hemicellulose content, the content of which is 7.9 wt.% in RF2, 7.2 wt.% in RF3 and 6.9 wt.% in RF4. Moreover, in four kinds of rice straw fibers, the highest lignin content reached 20.5 wt.% in RF2. Bleaching treatment significantly decreased the content of the lignin component, which decreased to 8.5 wt.% in RF3 and 2.6 wt.% in RF4 from 20.5 wt.% in RF2. Introduction of ozone was advantageous to the generation of HOO^−^ due to the reaction between H_2_O_2_ and oxygen radicals released from the ozone. In addition, the ozone also could degrade the residual lignin as described in Reference [29].

**Figure 1:**
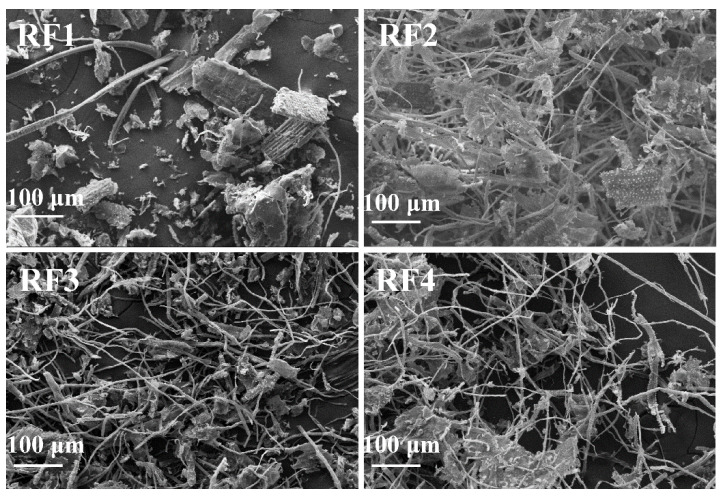
SEM images of rice straw fibers: RF1 (pristine rice straw fiber), RF2 (steam exploded rice straw fiber), RF3 (one bleached rice straw fiber), RF4 (twice beached rice straw fiber).

The length, diameter and the aspect ratio of the rice straw fibers are provided in Table 2. The SEM images of the rice straw fibers are shown in Figure 1. As shown in Table 2, the aspect ratios of RF2, RF3 and RF4 are 56.2, 97.8 and 71.6, respectively, while the precise aspect ratio of RF1 cannot be obtained. As shown in Figure 1, the morphology of the rice straw fibers included fragment, beam and fibrous structure. The untreated rice straw consists mainly of irregular fragments, which is why the exact aspect ratio cannot be obtained in RF1. Compared to RF1, the images of RF2 exhibit more fibrous shapes due to the autohydrolyzation and dissolution of the hemicelluloses and partial depolymerization of lignin [27,28]. For RF3 and RF4 with aspect ratio of more than 70, the SEM images shows clearer fibrous structure because different pretreatment processes lead to more cellulose content.

**Figure 2:**
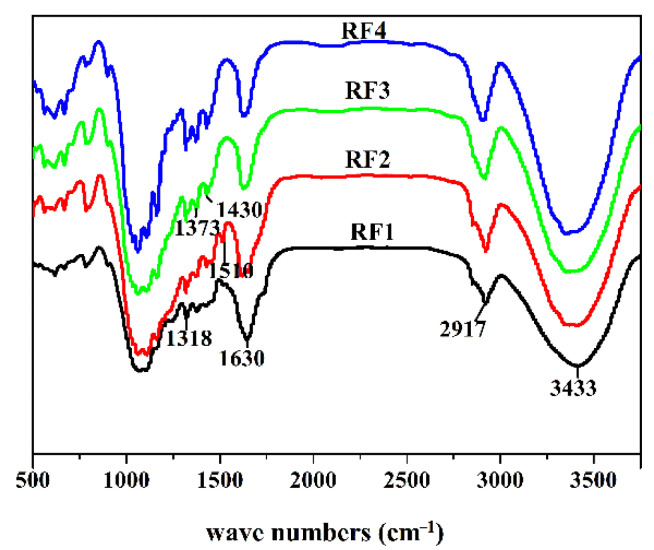
FTIR spectrums of the four rice straw fibers.

The FTIR spectrums of four rice straw fibers are shown in Figure 2. As can be seen from the figure, the broad characteristic absorption peak of the stretching vibration of -OH (3433 cm^−1^) was present in all samples, indicating presence of a large number of associated hydroxyl groups and extremely hydrophilicity. The sharp peak of the C-H bond stretching vibration was revealed at 2917 cm^−1^, embodying the saturated alkane chain in the chemical structure of plant fibers, such as the phenylpropane structure in the molecular structure of lignin, methylene and a small amount of methyl on the carbon shelf of cellulose and hemicellulose molecules [17]. The characteristic peak at 1630 cm^−1^ represents the absorption peak of water due to excellent hydrophilicity of the pure cellulose [24]. The small band at 1510 cm^−1^ represents the skeleton vibration of the benzene ring within fiber [30,31], which was most obvious in the sample of RF2, relatively weak in the sample of RF1 and could hardly be seen in the sample of RF3 and RF4. The above results are in good agreement with the lignin content of each fiber in Table 1. The weak and small peaks at 1430, 1373 and 1318 cm^−1^ represent the C-H stretching vibration peak, C-H bending vibration peak and O-H bending vibration peak, respectively. In addition, the absorption peak near 1054 cm^−1^ and shoulder peak on both sides are the typical absorption peaks of natural cellulose, and 783 cm^−1^ represents the C-H bending vibration peak outside the lignin benzene ring [32].

### 3.2. Influence of Rice Straw with Different Pretreatments on Heat of Hydration

**Figure 3:**
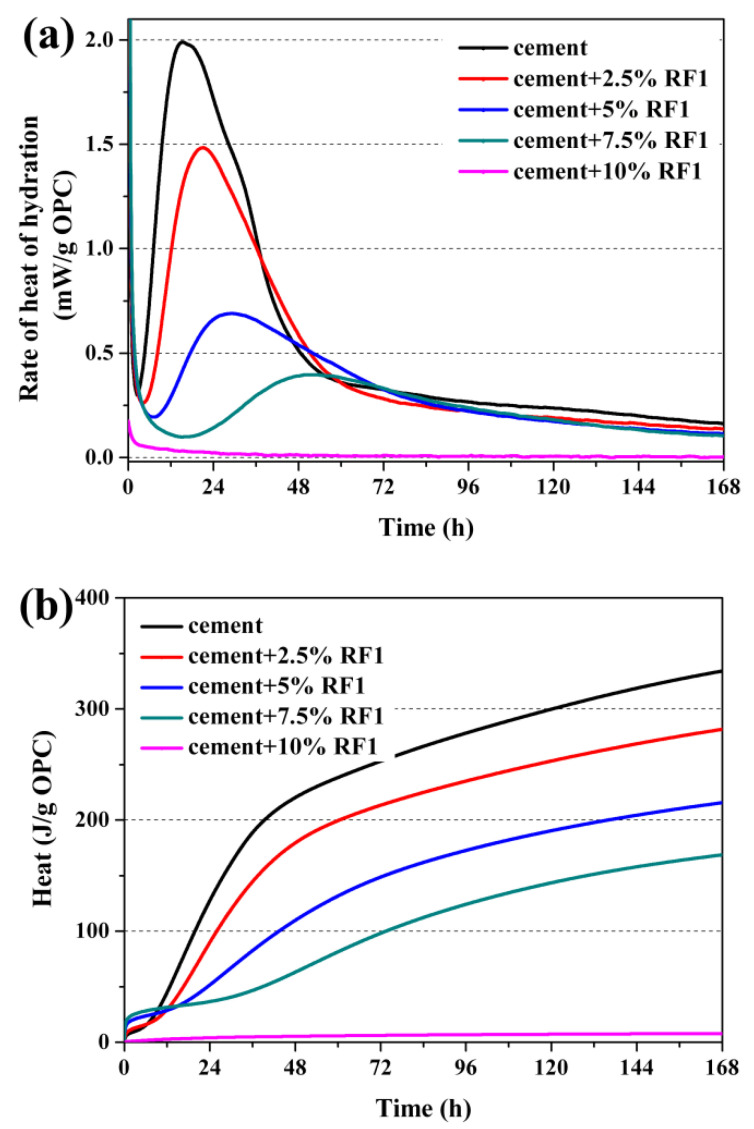
Heat of hydration of OPC-RF1: (**a**) rate of heat of hydration and (**b**) total hydration heat.

**Figure 4:**
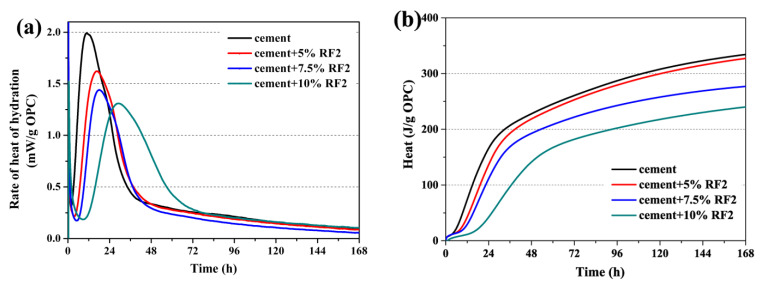
Heat of hydration of OPC-RF2: (**a**) rate of heat of hydration and (**b**) total hydration heat.

**Figure 5:**
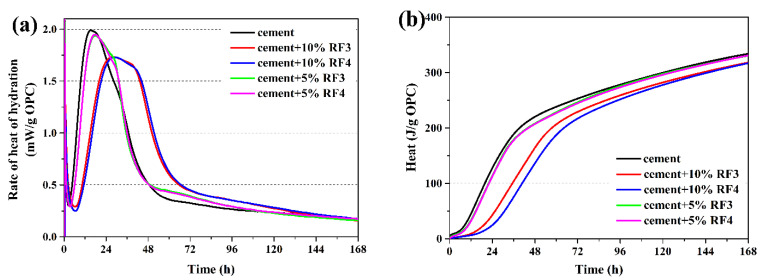
Heat of hydration of OPC-RF3/RF4: (**a**) rate of heat of hydration and (**b**) total hydration heat.

The heat of hydration curves of the composite samples with different rice straw fibers within 7 days are shown in Figure 3, Figure 4 and Figure 5. Table 5 shows the values of the total hydration heat in the first 7 days, the hydration time needed to reach the max rate of hydration heat and the max rate of hydration heat increment of the inhibited OPC.

In Figure 3, Figure 4 and Figure 5, the hydration process of the composite samples except 90OPC-10RF1 are divided into five stages of hydration, which is similar to that of the reference sample, as described by Taylor [33]. The sharp and short exothermic peak immediately appeared after addition of water, which was the first stage due to the partial dissolution and initial reaction on the surface of the cement particles. Then, rate of heat of hydration decreased rapidly, which was the second stage, the induction period of hydration. At this stage, the exothermic rate is very low and the hydration reaction is relatively slow. After holding for a period, the hydration reaction reaccelerated and entered the third stage, the acceleratory period, in which the main reaction first began to occur rapidly and reached the second exothermic peak. This takes place during the acceleratory period. The fourth stage was the decelerator period, in which the rate of heat of hydration evolution rate decreased with the reduction in active phases. Finally, the curves of hydration heat release achieved a period of slow, continued reaction, which is the fifth stage. For the sample 90OPC-10RF1, a brief and small exothermic peak appeared when the solid raw materials contacted with water, then a long dormant period was followed until the test was ended.

According to the hydration curves of Figure 3a and the data of Table 5, the addition of the original rice straw (RF1) significantly slowed hydration of cement, and the delay degree increases with the increase in fiber content. The hydration almost stops when OPC is replaced by 10 wt.% RF1. According to Figure 3a, the maximum rate of heat of hydration approached 2.0 mW/g at 15.2 h for the reference sample. In the composite samples filled with 2.5, 5.0 and 7.5 wt.% RF1, the maximum rate of heat of hydration was 1.5 (at 21.0 h), 0.7 (at 29.2 h) and 0.4 mW/g (at 52.9 h), respectively. As shown in Figure 3b and Table 5, as compared with 334.2 J of the reference sample, the cumulative hydration heat of per unit OPC with 2.5, 5, 7.5 and 10 wt.% RF1 decreased to 281.7, 215.6, 168.7 and 7.9 J, respectively. Due to addition of 2.5–10 wt.% RF1, the cumulative hydration heat of per unit OPC decreased to 2.4–84.3% of that of the reference specimen in 7 days.

Combining Figure 4a with Table 5, the influence of RF2 on hydration of OPC is similar to RF1. The incorporation of RF2 delays the hydration of OPC, and the more RF2 content is added, the higher the delay degree will be. In addition, when the composite samples are filled with 5.0, 7.5 and 10 wt.% RF2, the maximum rate of heat of hydration was 1.6 (at 16.7 h), 1.4 (at 18.1 h) and 1.3 mW/g (at 29.1 h), respectively. According to Figure 4b and Table 3, the cumulative hydration heat of per unit OPC filled with 5, 7.5 and 10 wt.% RF2 was 97.8%, 82.9% and 71.8% of that of the reference sample within 7 days, respectively.

As shown in Figure 5 and Table 5, RF3 and RF4 have almost the same effect on the hydration of OPC; the cumulative hydration heat of per unit OPC of 95OPC-5RF3, 90OPC-10RF3, 95OPC-5RF4 and 90OPC-10RF4 was 98.9%, 95.2%, 99.0% and 94.9% of that of the reference sample within 7 days, respectively. Moreover, compared with RF1 and RF2, RF3 and RF4 have less influence on the hydration of OPC.

Through the above analysis, it can be found that RF not only reduces the hydration heat of OPC but also delays the main hydration reactions of OPC. The reduction degree of hydration heat and the decreased hydration reactions increase with the increase in RF content. In case of the same RF content, addition of 5 wt.% RF1, RF2, RF3 and RF4 results in the total hydration heat evolution within 7 days decreasing to 64.5%, 97.9%, 98.9% and 99.0% of the reference sample, respectively, while addition of 10 wt.% RF1, RF2, RF3 and RF4 leads to the total hydration heat evolution within 7 days decreasing to 2.4%, 71.8%, 95.2% and 94.9% of the reference sample, respectively. The above results are attribute to variation of the contents of main components of the fibers. Steam explosion destroys the water extractives and removes the hemicellulose component of rice straw fiber [34]. Due to the partial water solubility of hemicellulose, its existence has a negative effect on the setting of cement [35]. According to Bilba et al. [36], the hydration delay of OPC can also be due to lignin hydrolysis. 

### 3.3. Influence of Rice Straw with Different Pretreatments on Inhibitory Index

The inhibitory index of the composite samples was calculated by the values of the total hydration heat in the first 7 days, the hydration time needed to reach max rate of hydration heat and the max rate of hydration heat increment of the inhibited OPC, as shown in Table 5. The inhibitory grades for the different RF-OPC samples were determined according to the study of Okino et al. [20], as shown in Table 6. From Table 5, the pretreated rice straw fibers have better compatibility with OPC than the untreated rice straw fibers. The inhibitory index achieves the extreme inhibitory grade when 7.5 and 10 wt.% RF1 are added, and the inhibitory index belongs to the moderate inhibitory grade when 5 wt.% RF1 and 10 wt.% RF2 are added. Except for the above samples, the inhibitory indexes of 97.5OPC-2.5RF1, 95OPC-5RF2, 92.5OPC-7.5RF2, 95OPC-5RF3, 90OPC-10RF3, 95OPC-5RF4 and 90OPC-10RF4 are in the low inhibitory grade.

The results for the inhibitory index of different RF-OPC samples are directly related to contents of hemicellulose, lignin and cellulose [20,21]. As shown in Table 4 and Table 5, comparing 90OPC-10RF1 with 92.5OPC-7.5RF2, cellulose, lignin and hemicellulose contents of the two samples are 3.56, 1.68, 2.05 and 3.80, 1.54, 0.60 wt.%, respectively. However, the inhibitory grades of fiber and OPC are extreme inhibitory and low inhibitory, respectively. Similarly, for 95OPC-5RF1 with moderate inhibitory grade and 95OPC-5RF2 with low inhibitory grade, their cellulose, lignin and hemicellulose contents are 1.78, 0.84, 1.03 and 2.53, 1.03, 0.40 wt.%, respectively.

**Figure 6:**
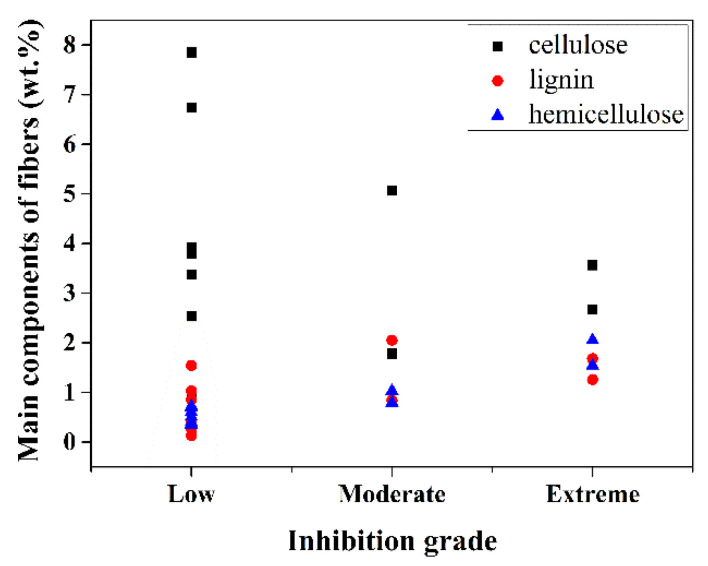
Diagram of inhibition grade versus cellulose, lignin and hemicellulose in the RF-OPC samples.

A diagram of the inhibition grade versus the content of hemicellulose, lignin and cellulose is shown in Figure 6. The figure shows that the content and change of hemicellulose had the most significant effect on the inhibition grade. The inhibitory grade reached the extreme grade when the hemicellulose content was 1.54 and 2.05 wt.%, while the hydration of OPC was stopped when the hemicellulose content was 2.05 wt.%. Thomas and Birchall [37] deem that the extractives of plant fibers adsorb Ca^2+^ ions, slowing the OPC hydration. Therefore, the extractives of plant fibers are involved in free calcium sequestration in the process of hydration of OPC and change the formation process of the hydration products, leading to composites with lower mechanical properties. Thus, the influence of RF1 on the hydration of OPC is in agreement with results reported by Olorunnisola [38] for rattan. Kochova et al. [39] investigated the effect of pure saccharides and the leachates of six natural fibers on the hydration of OPC, and they found that glucose, mannose and xylose in fiber leachates slowed down the cement hydration. In addition, cellulose is the least influential factor in the hydration of cement among the three main components, as shown in Figure 6, because 90OPC-10RF3 and 90OPC-10RF4 are in the low inhibitory grade range even if cellulose content reaches 6.74 and 7.85 wt.%, respectively. The influence of lignin on the hydration of OPC is between cellulose and hemicellulose. As far as 90OPC-10RF2 and 90OPC-10RF3 are concerned, the hemicellulose content is almost the same. However, the lignin content of 2.05 and 0.85 wt.% leads to moderate inhibitory index and low inhibitory index, respectively.

### 3.4. XRD Analysis of the Composite Samples

**Figure 7:**
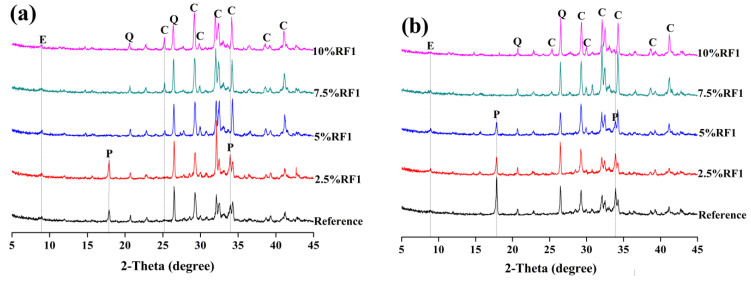
XRD patterns of OPC-RF1 at (**a**) 3 days and (**b**) 7 days (E—ettringite, Q—quartz, C-C_3_S and C_2_S, P—portlandite).

**Figure 8:**
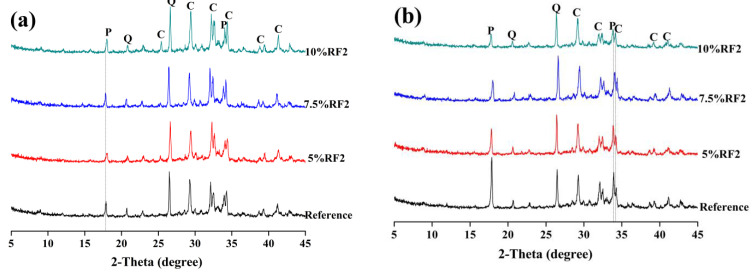
XRD patterns of OPC-RF2 at (**a**) 3 days and (**b**) 7 days (E—ettringite, Q—quartz, C-C_3_S and C_2_S, P—portlandite).

**Figure 9:**
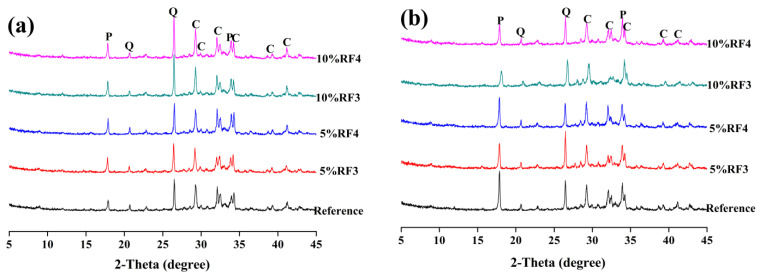
XRD patterns of OPC-RF3 and OPC-RF4 at (**a**) 3 days and (**b**) 7 days (E—ettringite, Q—quartz, C-C_3_S and C_2_S, P—portlandite).

Figure 7 and Figure 8 and Figure 9 reveal the XRD patterns of the composite samples with RF1, RF2, RF3 and RF4 after 3 and 7 days. As shown in the XRD patterns of the composite samples with RF1 in Figure 7, addition and content of RF1 significantly affected the hydration of OPC. After 3 days, the diffraction peak of calcium hydroxide was not found in 95OPC-5RF1, 92.5OPC-7.5RF1 or 90OPC-10RF1 samples, while the diffraction peak of calcium hydroxide still did not appear in 92.5OPC-7.5RF1 and 90OPC-10RF1 samples after 7 days, although 92.5OPC-7.5RF1 has acceleration and deceleration periods in the hydration heat curves of Figure 3a. The results indicate that a certain amount of RF1 delay, even stopping the hydration of OPC, which is consistent with the results of hydration heat. Compared with the effect of RF1, RF2 has a much smaller effect on the hydration of OPC. At 3 days, the diffraction peak of calcium hydroxide can be detected in all the samples, including the sample with 10 wt.% RF2 content as shown in Figure 8a. However, the XRD results indicated that the change of RF2 content significantly affects the hydration process of OPC according to the change trend of the intensity of the diffraction peak of calcium hydroxide in Figure 8b. Comparing the XRD patterns of Figure 9 with those of Figure 7 and Figure 8, it can be observed that addition of RF3 and RF4 has less effect on the hydration of OPC than addition of RF1 and RF2. The reason is that a large amount of hemicellulose and lignin in rice straw RF1 prolongs the hydration induction period of cement, making the cement hydration stop [36].

### 3.5. SEM Analysis of the Composite Samples

**Figure 10:**
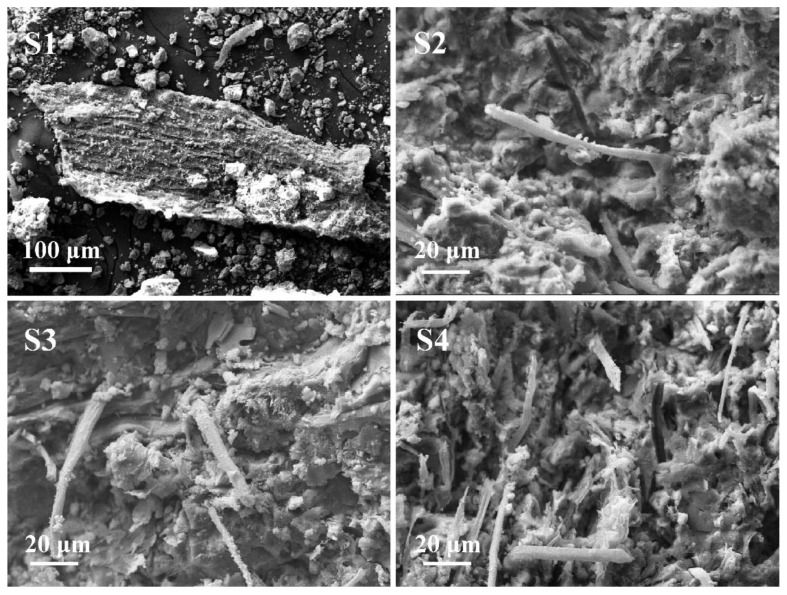
SEM images of fracture surface of the composite samples curing for 7 days: S1 (90OPC-10RF1), S2 (90OPC-10RF2), S3 (90OPC-10RF3), S4 (90OPC-10RF4).

In order to understand the distribution of the four rice straw fibers in the cement matrix and the combination between different straw fibers and the cement matrix, the morphological characteristics of the fracture of the composite samples with 10% fiber content were observed using a scanning electron microscope. Figure 10 shows the SEM images of the fracture of the composite samples after 7 days of hydration aging.

As shown in Figure 10, for the untreated RF/cement-based composite S1 (90OPC-10RF1), only dispersed cement particles and rice straw fiber were observed in the SEM image, and no cohesion between the fiber and the cement was found, indicating that the cement in the composites does not set or harden. In the SEM image of S2 (90OPC-10RF2), it can be observed that there were few pulled out fibers and fragmented fiber structure, indicating that the toughness of the fiber is poor, and the fiber is broken when the sample is stressed. In the SEM images of S3 (90OPC-10RF3) and S4 (90OPC-10RF4), some holes and pulled out fibers are observed. Compared with RF1 and RF2, RF3 and RF4 possess smaller amounts of amorphous substances hemicellulose and lignin, resulting in higher elastic modulus [40] and better adhesion with cement matrix.

### 3.6. Mechanical Properties Analysis of the Composite Samples

**Figure 11:**
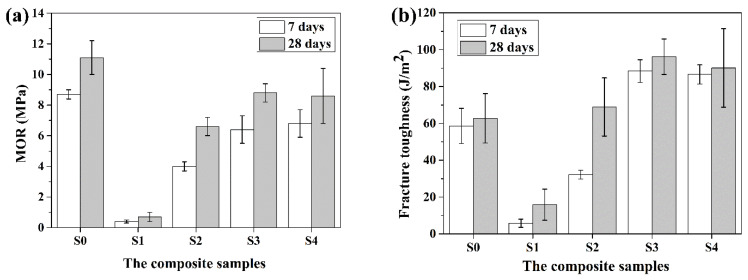
The mechanical properties of the samples: (**a**) maximum flexural strength, (**b**) fracture toughness. S0 (OPC), S1 (90OPC-10RF1), S2 (90OPC-10RF2), S3 (90OPC-10RF3), S4 (90OPC-10RF4).

Figure 11 shows the maximum flexural strength (MOR) and fracture toughness of the composite samples with 10 wt.% RF1, RF2, RF3 and RF4 after 7 and 28 days, respectively, and a comparison with the reference sample cement paste (S0). As shown in Figure 11a, adding 10 wt.% RF1, RF2, RF3 and RF4 causes about a 95.4%, 54.0%, 26.4% and 21.8% reduction in the maximum flexural strength values of composites compared to the reference cement paste of 7 days. A similar behavior pattern is revealed for the composite samples of 28 days; compared with the reference samples, the maximum flexural strength of the composites with 10 wt.% RF1, RF2, RF3 and RF4 declines by about 84.7%, 40.5%, 20.7% and 22.5%. Moreover, the MOR values of the samples at 28 days are higher than those of 7 days. The possible reasons for the above phenomenon are as follows. Firstly, in combination with the hydration heat and XRD analysis result, addition of 10 wt.% rice straw fibers causes a delay of the hydration degree of cement. The hydration degree of cement is related to the strength of the cement, which increases with the hydration degree of the cement. This is consistent with the description of Bilba et al. [36], which suggests that hydrolysis of lignin, an amorphous substance of fiber composition, and the dissolution of hemicellulose delays or even stops the hydration process of cement, affecting the mechanical properties of cement-based composites. As a result, the maximum flexural strength of the samples with RF1 and RF2 is only 0.4 and 4.0 MPa at 7 days. Secondly, for the samples with RF3 and RF4, a small quantity of fibers could not distribute uniformly in the matrix, and some areas of the samples were without fiber, which could lead to initial cracks grown in these areas [41].

Figure 11b shows that the introduction of RF1 and RF2 results in the decrease in the fracture toughness of the samples, while adding RF3 and RF4 increases the fracture toughness of the samples. Compared with the samples without fibers, introduction of RF1 and RF2 results in the fracture toughness values of 7 days decreasing 90.1% and 45.1%, and the addition of RF3 and RF4 leads to the fracture toughness of 7 days increasing 50.9% and 47.8%. Combined with the hydration heat analysis, RF1 and RF2 have a great effect on the cement hydration, so the influence of cement hydration degree is dominant. For the samples with RF3 and RF4, when the samples are subjected to external force, the stress is transferred to the fiber from the cement matrix. When the fibers are pulled out from the matrix, friction is generated, which consumes part of the energy and improves the fracture toughness of the sample [42].

## 4. Conclusions

In this study, compatibility between rice straw fibers with different pretreatments and OPC was evaluated by measuring the hydration process and hydration heat of OPC with different types and contents of rice straw fibers. The following conclusions are drawn:

- Steam explosion treatment of rice straw fibers removes most hemicellulose, while the action of bleaching treatment gets rid of lignin.
- The hydration process of the composite samples is similar to that of OPC except the 90OPC-10RF1 sample. In addition, the hydration heat of the composite samples is affected by both the pretreatment degree of rice straw fiber and fiber content.
- The inhibitory index value of different RF-OPC samples is directly related to hemicellulose and lignin content of rice straw fibers. In particular, the content of hemicellulose significantly affects cement hydration. When the hemicellulose content was 1.54 wt.% in the composite sample, the inhibitory index reached the extreme grade, and the hydration of OPC stopped when the hemicellulose content was 2.05 wt.%.
- Combining XRD and SEM analysis of the different rice straw fibers filled cement-based composite samples, better compatibility and adhesion were observed between the pretreated fibers and OPC due to removal of hemicellulose and lignin.

In this work, the compatibility between rice straw and OPC was studied. In the next step, influence of other crop straw and plant fibers on the hydration of OPC can be considered, so as to provide more theoretical research for the application of crop straw in building materials.

## Figures and Tables

**Table 1:** The chemical components of the rice straw fibers.

Sample	Components (wt.%)					
	Cellulose	Lignin	Hemicellulose	Ash	Moisture	Other
RF1	35.6	16.8	20.5	15.1	12.0	0.0
RF2	50.6	20.5	7.9	12.5	8.5	0.0
RF3	67.4	8.5	7.2	8.5	8.3	0.1
RF4	78.5	2.6	6.9	3.1	8.9	0.0

**Table 2:** The properties of rice straw fibers.

Fiber Type	Length (μm)		Diameter (μm)		Aspect Ratio
	Average ^a^	S ^b^	Average ^a^	S ^b^	
RF1	--	--	--	--	--
RF2	354.2	112.0	6.3	1.6	56.2
RF3	577.5	154.7	5.9	1.6	97.8
RF4	472.6	85.9	6.6	2.0	71.6

^a^ Avera ge length and diameter obtained from 30 fibers of each type. ^b^ Standard deviation of each sample: S = ($\sum {\left(x-\bar{x}\right)}^{2}/\left(n-1\right)$) ^1/2^, n = 30.

**Table 3:** The chemical components of OPC.

Components (wt.%)	CaO	SiO_2_	Al_2_O_3_	SO_3_	Fe_2_O_3_	K_2_O	MgO	TiO_2_	Na_2_O	P_2_O_5_	BaO	Cl	Other
cement	62.4	18.9	4.83	4.73	2.85	0.81	2.39	0.47	0.41	0.35	0.14	0.01	1.71

**Table 4:** Mix formulation of the composite samples with rice straw fibers.

Mix	Proportion (wt.%)				
	OPC	Straw Fiber	Cellulose	Lignin	Hemicellulose
Reference	100	0	0	0	0
97.5OPC-2.5RF1	97.5	2.5	0.89	0.42	0.51
95OPC-5RF1	95	5	1.78	0.84	1.03
92.5OPC-7.5RF1	92.5	7.5	2.67	1.26	1.54
90OPC-10RF1	90	10	3.56	1.68	2.05
95OPC-5RF2	95	5	2.53	1.03	0.40
92.5OPC-7.5RF2	92.5	7.5	3.80	1.54	0.60
90OPC-10RF2	90	10	5.06	2.05	0.79
95OPC-5RF3	95	5	3.37	0.43	0.36
90OPC-10RF3	90	10	6.74	0.85	0.72
95OPC-5RF4	95	5	3.93	0.13	0.35
90OPC-10RF4	90	10	7.85	0.26	0.69

**Table 5:** Inhibitory index of the composite samples.

Mix	Q (J/g)	Q’ (J/g)	H (h)	H’ (h)	S (J/g/h)	S’(J/g/h)	Inhibitory Index (%)	Grade
97.5OPC-2.5RF1	334.2	281.7	15.2	21.0	0.23	0.13	2.606	Low
95OPC-5RF1	334.2	215.6	15.2	29.2	0.23	0.05	25.580	Moderate
92.5OPC-7.5RF1	334.2	168.7	15.2	52.9	0.23	0.02	112.679	Extreme
90OPC-10RF1	334.2	7.9	15.2	Infinite	0.23	0.01	Infinite	Extreme
95OPC-5RF2	334.2	327.2	15.2	16.7	0.23	0.20	0.027	Low
92.5OPC-7.5RF2	334.2	240.1	15.2	18.1	0.23	0.18	1.168	Low
90OPC-10RF2	334.2	240.1	15.2	29.1	0.23	0.10	14.554	Moderate
95OPC-5RF3	334.2	330.4	15.2	18.0	0.23	0.20	0.027	Low
90OPC-10RF3	334.2	318.2	15.2	28.1	0.23	0.12	1.943	Low
95OPC-5RF4	334.2	330.9	15.2	17.6	0.23	0.20	0.020	Low
90OPC-10RF4	334.2	317.1	15.2	29.0	0.23	0.12	2.222	Low

**Table 6:** Inhibitory index grade used to RF-OPC compatibility [20].

Inhibitory Index (%)	Grade
I < 10	Low inhibitory
I = 10–50	Moderate inhibitory
I = 50–100	High inhibitory
I > 100	Extreme inhibitory

## Data Availability

Not applicable.
